# Osteoimmunology: interactions of the bone and immune system

**DOI:** 10.3389/fimmu.2026.1925819

**Published:** 2026-09-22

**Authors:** Qinling Mo, Yunhe Jiang, Xiaoguang Han, Changwei Li, Guoqing Tang

**Affiliations:** 1 Department of Oncology, Kunshan Hospital of Chinese Medicine, Affiliated Hospital of Yangzhou University, Suzhou, Jiangsu, China; 2 Department of Central Laboratory, Kunshan Hospital of Chinese Medicine, Affiliated Hospital of Yangzhou University, Suzhou, Jiangsu, China; 3 Department of Clinical Laboratory, Kunshan Hospital of Chinese Medicine, Affiliated Hospital of Yangzhou University, Suzhou, Jiangsu, China; 4 Department of Spine Surgery, Beijing Jishuitan Hospital, Beijing, China; 5 Department of Orthopedics, Shanghai Key Laboratory for Prevention and Treatment of Bone and Joint Diseases, Shanghai Institute of Traumatology and Orthopedics, Ruijin Hospital, Shanghai Jiao Tong University School of Medicine, Shanghai, China; 6 Department of Orthopedics, Kunshan Hospital of Chinese Medicine, Affiliated Hospital of Yangzhou University, Suzhou, Jiangsu, China

**Keywords:** bone marrow microenvironment, bone metabolism, inflammation, musculoskeletal disorders, osteoimmunology

## Abstract

Osteoimmunology represents an emerging interdisciplinary domain that investigates the reciprocal crosstalk between the skeletal and immune compartments, which serves as the core immunological basis for the pathogenesis of diverse musculoskeletal disorders. This bidirectional communication is facilitated by a shared microenvironment and common molecular mediators. This review systematically synthesizes the regulatory effects of T cells, B cells, and macrophages on bone homeostasis. Their modulatory actions are predominantly executed via proinflammatory cytokines and canonical signaling cascades, with particular emphasis on the RANKL/RANK/OPG axis. In parallel, we highlight that bone lineage cells (e.g., osteoblasts and osteoclasts) are not merely passive targets of immune regulation. Instead, they actively participate in immune cell development and function by secreting cytokines. Together, these interactions form an integrated and dynamic regulatory balance. Under physiological conditions, this finely tuned bone-immune crosstalk maintains bone-immune homeostasis. Its disruption, however, contributes to the pathogenesis of typical musculoskeletal pathologies, including rheumatoid arthritis, osteoporosis, and tumor-associated bone metastasis. Adopting an integrated perspective, this review integrates the shared regulatory mechanisms and microenvironment-dependent features of osteoimmune interactions. It provides a theoretical framework for understanding osteoimmune imbalance in musculoskeletal disorders and for developing precise therapeutic strategies.

## 1 Introduction

The skeletal system is a highly specialized organ system, traditionally appreciated for its roles in mechanical support, protection, and locomotion. However, the skeleton also participates in sophisticated inter-organ communication with distant tissues, including the immune, endocrine, and central nervous systems. Within this systemic network, the osteoimmune axis serves as a paradigm. In this framework, bone-derived factors act not merely as local paracrine signals but as endocrine messengers that integrate immune status with whole-body metabolic homeostasis (1–3).

The skeletal and immune systems are closely interconnected in terms of anatomical localization, cellular origin, and molecular regulation. The bone marrow constitutes the shared anatomical microenvironment for both systems. This shared niche not only serves as the primary site for hematopoiesis and immune cell differentiation, but also as the key region where continuous bone remodeling occurs. Traditionally, these two systems were considered relatively independent physiological units. Nevertheless, the conventional perspective was substantially altered when Arron and Choi formally put forward the concept of osteoimmunology in the early 2000s (4). This emerging interdisciplinary field is dedicated to examining the multifaceted interplay between the skeletal system and the immune system (5). Over the past few years, a growing collection of evidence has gradually uncovered extensive molecular cross-talk and reciprocal functional reliance between these two systems.

The fundamental paradigm of this field is predicated on the bidirectional dialogue between bone-associated cells (for instance, osteoblasts, osteoclasts, and osteocytes) and immunocytes (such as T cells, B cells, and dendritic cells). The crosstalk between these cells is governed by overlapping molecular components, ranging from cytokines and chemokines to transcription factors, receptors, and signaling routes, as cited in the literature (3, 6–8). On the immune side, cytokines released by these cells can directly impinge upon the differentiation, functionality, and longevity of osteoblasts and osteoclasts, thus playing a pivotal role in maintaining bone metabolic homeostasis. On the other hand, bone lineage cells secrete signaling molecules that modulate immune cell development, activation, and function. This finely tuned “bone–immune crosstalk” cooperatively maintains physiological homeostasis under normal conditions (9, 10). Once disrupted, however, it can drive the initiation and progression of a wide range of diseases.

Over the past two decades, osteoimmunology research has evolved from the initial conceptual proposition into in-depth dissection of microscale molecular mechanisms, with continuous advancement toward clinical translation. Substantial evidence demonstrates that osteoimmune imbalance plays a central role in numerous diseases, including rheumatoid arthritis, osteoporosis, osteoarthritis, periodontitis, and tumor-associated bone disorders. The establishment of osteoimmunology has not only greatly expanded our understanding of disease pathogenesis, but has also provided an important theoretical foundation for novel therapies targeting immune-bone networks. Despite this progress, key challenges remain. These objectives involve delineating the changing contributions of immune cells to bone lesions, establishing the molecular mechanisms responsible for counterbalancing pro- and anti-inflammatory activities, and bridging osteoimmunology research with clinical applications.

Therefore, it is necessary to re-examine osteoimmune regulatory networks from a multi-level perspective encompassing cells, molecules, microenvironments, and disease phenotypes. Herein, we provide a thorough overview of the latest developments within the field of osteoimmunology. Particular attention is paid in this review to the molecular bases of immune-directed bone remodeling, the regulatory inputs delivered by bone cells to the immune system, the bridging functions of key cytokines and signaling conduits, as well as the clinical significance of osteoimmune perturbation. The ultimate aim is to determine the feasibility of applying these findings to the design of precise clinical interventions.

## 2 Literature search strategy

This narrative review was based on a literature search of PubMed and Web of Science (2000–2026) using keywords related to osteoimmunology, bone-immune crosstalk, and cellular senescence. Additional citations were retrieved by cross-referencing the bibliographies of key articles.

## 3 Regulation of bone metabolism by immune cells

Both directly and indirectly, immune cells modulate the differentiation trajectories and functional phenotypes of osteoblasts and osteoclasts. Key mediators include bone marrow-resident T and B lymphocytes and macrophages, which exert their effects through secreted cytokines and membrane-bound signaling molecules. Consequently, immune cells possess the dual capacity to both support the maintenance of bone homeostasis and, conversely, to precipitate substantial bone loss. A classic illustration of this regulatory effect is the RANKL/RANK/OPG pathway, which functions as the principal molecular connector between immune activation and skeletal metabolism (8, 11, 12). Dysregulated immune activation perturbs the equilibrium between bone formation driven by osteoblasts and bone resorption executed by osteoclasts (13, 14). Therefore, understanding immune-mediated regulation of bone metabolism under both physiological and pathological conditions is fundamental. Appreciation of this relationship is vital, both for interpreting the pathological basis of osteoporosis and rheumatoid arthritis (RA), and for pioneering new immune-oriented therapeutic strategies. This section provides a comprehensive account of how three major immune populations—T cells, B cells, and macrophages—contribute to the modulation of bone turnover, with emphasis on their respective functions and signaling mechanisms.

### 3.1 Roles of T lymphocytes

Bone remodeling, a dynamic event sustained by the balanced coupling of bone deposition and resorption, is additionally regulated by immune cells within the bone marrow microenvironment, with T lymphocytes playing an especially critical part. As core constituents of immunity, T cells fulfill dual roles: they participate in immune surveillance while concurrently exerting considerable control over bone remodeling, an effect mediated by their regulation of osteogenic and osteoclastogenic cell differentiation and function (15). Under conditions of chronic inflammation and in bone-associated diseases, aberrant activation of T cells can induce bone damage (8, 16–18). T lymphocytes orchestrate osteoclast development and functional activity by secreting a broad array of cytokines, with effects that may be direct or indirect (19–21). Corroborating this perspective, Srivastava and associates introduced “immunoporosis” (22), a term that accentuates the critical contribution of immunological imbalance to the pathogenesis and aggravation of osteoporosis.

#### 3.1.1 Dual roles of T lymphocytes in bone homeostasis

T lymphocytes exert a dual modulatory effect on bone turnover, whose ultimate direction is determined by the interplay of activation stage, subset heterogeneity, and microenvironmental signals. During inflammation or pathology, activated T cells generate abundant RANKL and TNF-α (20, 22–25), which act as key promoters of osteoclast-mediated bone destruction (26).Conversely, certain T cell subsets exhibit bone-protective effects under physiological or immune homeostasis conditions. For example, regulatory T cells (Tregs) can directly inhibit osteoclastogenesis by suppressing the production of RANKL and macrophage colony-stimulating factor (M-CSF) (27). CD8^+^ T cells can suppress osteoclast formation through the secretion of osteoprotegerin (OPG) and interferon-γ (IFN-γ) (28, 29). However, when CD8^+^ T cells undergo functional exhaustion, their ability to inhibit osteoclastogenesis is diminished.

#### 3.1.2 Subset-specific functions of CD4^+^ and CD8^+^ T cells

CD4 and CD8 represent the canonical markers that discriminate between the two main functional arms of T cells. Both subsets are indispensable components of the immune system, having well-established, non-overlapping roles while simultaneously operating in a closely coordinated manner (30). Naive CD4^+^ T cells differentiate into multiple subsets, including Th1, Th2, Th9, Th17, Th22, Tregs, and follicular helper T cells (TFH). These subsets play distinct roles in immune regulation and bone metabolism (**Figure 1**) (22, 31–34).

**Figure 1:**
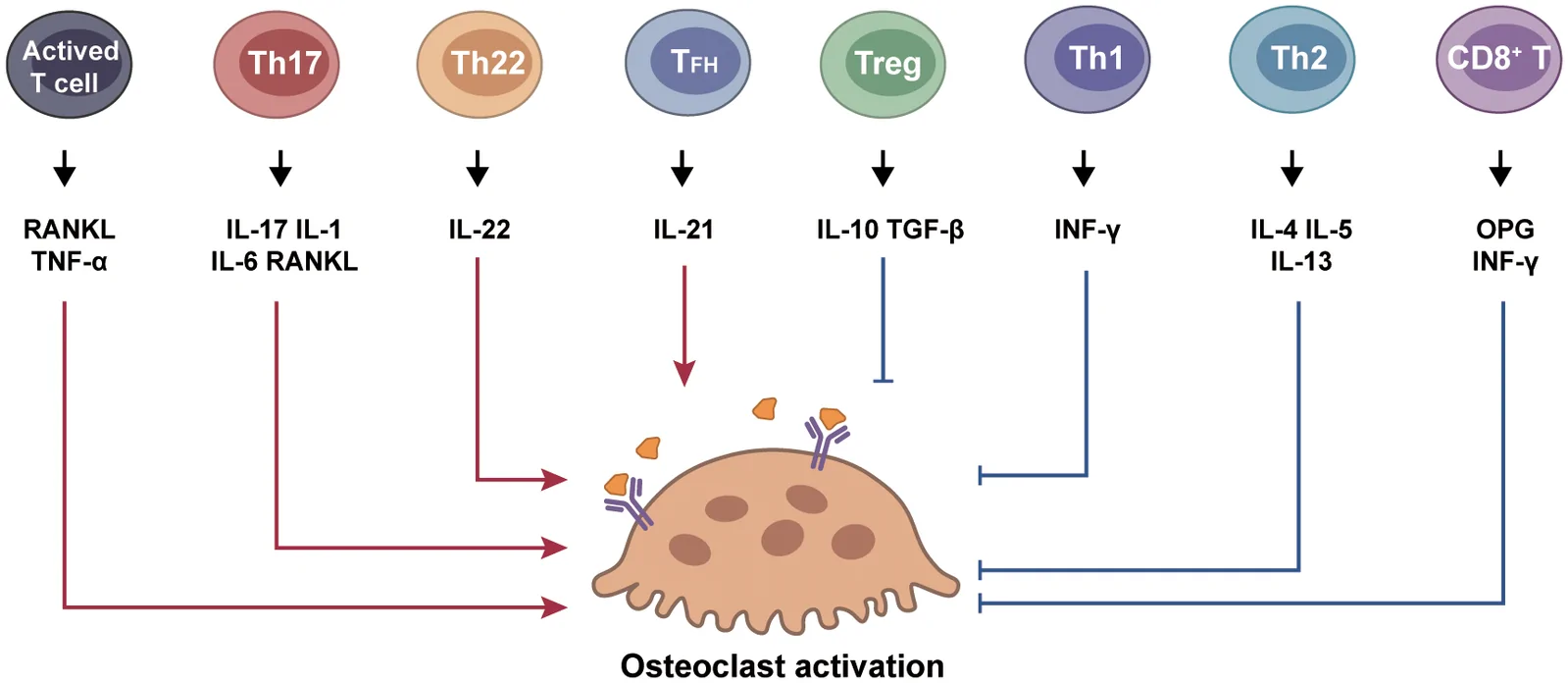
Bidirectional regulation of osteoclast activation by distinct T cell subsets and their secreted factors. In this image, the osteoimmune network is systematically illustrated with an emphasis on how T-cell subsets and other adaptive immune cells regulate osteoclast activation via cytokines. Osteoclasts, serving as the major bone-resorbing effector cells, become pathologically hyperactive in conditions such as osteoporosis and rheumatoid arthritis, a state that lies at the core of their pathogenesis. Activated T cells, Th17, and Th22 promote osteoclast progenitor differentiation and resorptive activity through RANKL, TNF-α, IL-17, and IL-22, whereas Tregs, Th1, and Th2 exert inhibitory effects via IL-10/TGF-β, IFN-γ, and IL-4/IL-5/IL-13, respectively. Moreover, Tregs can inhibit osteoclastogenesis by suppressing the production of RANKL and M-CSF. These anti-osteoclastogenic factors operate by diminishing the expression of critical mediators or by blocking downstream effector pathways. CD8^+^ T cells reinforce this protective regulation by releasing OPG, which acts as a soluble RANKL decoy that prevents RANKL from engaging RANK on osteoclast precursors, thus curtailing RANKL-triggered osteoclastogenesis.

In general, Th1 and Th2 cells are predominantly associated with protective effects on bone. Th1 cells contribute to the mitigation of osteoporosis primarily through the release of IFN-γ, a cytokine that impedes osteoclast differentiation and curtails bone resorptive activity (18, 35). Th2 cells exert a dual protective effect on bone: they secrete IL-4, IL-5, and IL-13 to hinder osteoclastogenesis (36–38), while concurrently diminishing bone loss through substantial downregulation of the RANKL/OPG ratio (39). Conversely, Th17 cells are broadly acknowledged as principal instigators of inflammatory osteolysis. They drive osteoclast formation and initiate bone resorption by producing high levels of pro-osteoclastogenic mediators, such as IL-1, IL-6, IL-17, RANKL, and TNF (21, 22, 40–42). The pathogenic involvement of Th17 cells has been firmly documented in both osteoporosis and rheumatoid arthritis (43–45). Th22 cells also contribute to osteoclast differentiation through the production of IL-22, and their role in local bone erosion has been identified in patients with rheumatoid arthritis (46, 47).

Tregs oppose osteoclast formation and maturation by secreting anti-inflammatory cytokines, notably IL-10 and TGF-β, thereby curbing bone resorption (14, 48–50). TFH cells, specialized in activating B cells via IL-21 secretion (51, 52), have been closely linked to bone destruction in autoimmune settings (53, 54). Th9 cells produce IL-9, and although emerging data suggest their potential participation in skeletal disorders such as rheumatoid arthritis (55), their direct contributions to bone metabolism await further clarification.

Presently, the majority of studies have been dedicated to CD4^+^ T-cell subsets, leaving the osteoimmunological role of CD8^+^ T cells largely underexplored. Nevertheless, CD8^+^ T cells can exert suppressive effects on osteoclast formation via the secretion of multiple soluble factors, such as OPG, IFN-γ, IL-10, and TGF-β. Their bone-sparing action has been validated using both *in vivo* and *in vitro* bone repair models (28, 29, 56, 57). Additionally, evidence indicates that CD4^+^ T cells also play a critical part in preserving gingival tissue homeostasis under chronic periodontitis conditions (57, 58). Overall, by participating in osteoimmune crosstalk, T cells emerge as a key factor in maintaining skeletal equilibrium. Their contributions are not confined to directly modulating osteoblast and osteoclast function, but are especially conspicuous in chronic inflammatory states associated with aberrant bone turnover.

#### 3.1.3 Molecular pathways underlying T cell–driven osteoimmune regulation

At the molecular scale, T cell-mediated control of bone turnover is accomplished through two key signaling axes. The RANKL/RANK/OPG pathway is central, with T-cell-derived RANKL engaging RANK on osteoclast precursors to drive osteoclast development and resorptive function (48, 59, 60), which in turn stimulate osteoclast development and resorptive function. In parallel, the S1P–S1PR1 axis contributes to osteoclastogenesis by redirecting CD4^+^ T cell differentiation and enhancing RANKL production in T cells (48, 61–63). These two signaling routes collectively form the major molecular link between T cells and bone. Their concerted action perturbs the normal balance of bone turnover and precipitates inflammation-mediated bone resorption.

T lymphocytes are indispensable components of osteoimmune regulation. They not only participate in the maintenance of bone homeostasis by promoting either bone resorption or bone formation through the secretion of relevant cytokines. Furthermore, T cells can be recruited via chemokine signaling pathways, such as CXCR3-mediated chemotaxis, to accelerate bone loss (64, 65). A key aggravating factor in pathological bone loss is the senescence of T cells induced by persistent inflammatory stimuli (66). Aging substantially alters T-cell function, with immunosenescence driving the expansion of CD28^−^ effector memory T cells and the production of pro-inflammatory cytokines that exacerbate bone loss (66, 67).Taken together, these molecular pathways form the essential framework for T-cell-mediated osteoimmune cross-talk, and their integrated actions are decisive for the development and advancement of inflammation-driven bone destruction.

### 3.2 Roles of B lymphocytes

#### 3.2.1 Physiological roles of B lymphocytes in bone homeostasis

B lymphocytes carry out three core immune functions: antibody production, antigen presentation, and cytokine secretion. In parallel, these cells constitute a major source of osteoprotegerin (OPG), accounting for approximately 64% of the OPG detected in the bone marrow (68). By acting as a decoy receptor for RANKL, OPG intercepts RANKL–RANK signaling, which in turn curtails osteoclastogenesis and bone resorption (69, 70). Substantial evidence indicates that under normal physiological conditions, B cells continuously release copious amounts of OPG (68), and this steady output is indispensable for the upkeep of bone homeostasis. This indispensable role is further validated by mouse studies showing that specific deletion of B cells leads to a sharp drop in OPG and concomitant skeletal deterioration (68), unequivocally demonstrating that B-cell-derived OPG exerts a critical bone-sparing effect *in vivo*.

#### 3.2.2 Pathological B cell activation and inflammatory bone destruction

B cells play an essential role in maintaining bone homeostasis, primarily as a key source of OPG. However, under pathological conditions, their activation and pro-inflammatory functions render them key drivers of osteoclastogenesis. Under inflammatory or autoimmune conditions, B cells undergo a functional shift. First, activated B cells can express RANKL (56, 71–73). Second, they secrete a variety of pro-inflammatory cytokines, such as TNF-α, IL-6, IL-1β, and IL-7 (74–76). These cytokines further stimulate RANKL expression and indirectly enhance osteoclast formation. Collectively, these mechanisms are closely associated with the initiation and progression of RA (74, 77–79).

Once B lymphocytes differentiate into plasma cells, they gain the ability to produce a range of autoantibodies, such as anti–citrullinated protein antibodies (ACPAs). These antibodies can assemble into immune complexes that activate the complement cascade and prompt macrophages and other immune cells to liberate large quantities of pro-inflammatory cytokines (80–83). This inflammatory milieu, in turn, indirectly fosters osteoclastic bone destruction. This mechanistic link is corroborated by clinical and experimental evidence showing that in rheumatoid arthritis, synovial tissues contain significantly higher concentrations of both RANKL and inflammatory cytokines derived from B cells, and that these elevations are closely associated with the severity of local bone erosion.

#### 3.2.3 Functional heterogeneity of B cell subsets in osteoimmune regulation

Emerging evidence indicates that B cells consist of functionally distinct subpopulations that exert divergent, and at times opposing, influences on bone homeostasis. Among these, regulatory B cells (Bregs) are characterized by their immunosuppressive capacity. In the context of specific inflammatory conditions or hormone-deficient states, Bregs have been shown to secrete anti-inflammatory cytokines, notably IL-10, TGF-β, and IL-35, thereby conferring protective effects on bone tissue (74, 84–86). In animal models of postmenopausal osteoporosis (PMOP), estrogen deficiency leads to a significant diminution in both the abundance and the functional competence of Bregs, which in turn shifts the RANKL/OPG balance toward a pro-resorptive state, and consequently aggravates skeletal loss (87, 88).

Age-associated B cells (ABCs) represent a recently recognized subset within the B-cell compartment. Notably, their increased frequency has been strongly associated with the development and aggravation of various autoimmune conditions. Mechanistically, ABCs have been shown to engage the ERK1/2 and JAK-STAT1 signaling cascades through TNF-α-mediated stimulation (76). This activation induces the subsequent activation of fibroblast-like synoviocytes (FLS). Ultimately, this ABC-driven cascade contributes to the inflammatory responses and joint bone destruction characteristic of rheumatoid arthritis. Aging also promotes the expansion of pro-inflammatory age-associated B cells (ABCs) that may contribute to bone loss in the elderly (76).

Collectively, B lymphocytes exhibit a dual regulatory role in bone metabolism (**Figure 2**). Under physiological conditions, they primarily maintain bone homeostasis through the secretion of OPG and exert bone-protective effects. However, upon aberrant immune activation, B cells transform into potent drivers of bone destruction. They promote osteoclastogenesis specifically by expressing RANKL and producing pro-inflammatory cytokines and autoantibodies. The ultimate impact of B cells on bone metabolism depends on local microenvironmental cues, their activation status, and subset composition. Collectively, these observations indicate that selective modulation of distinct B-cell subsets or their functional phenotypes could constitute a viable therapeutic approach for inflammatory bone disorders.

**Figure 2:**
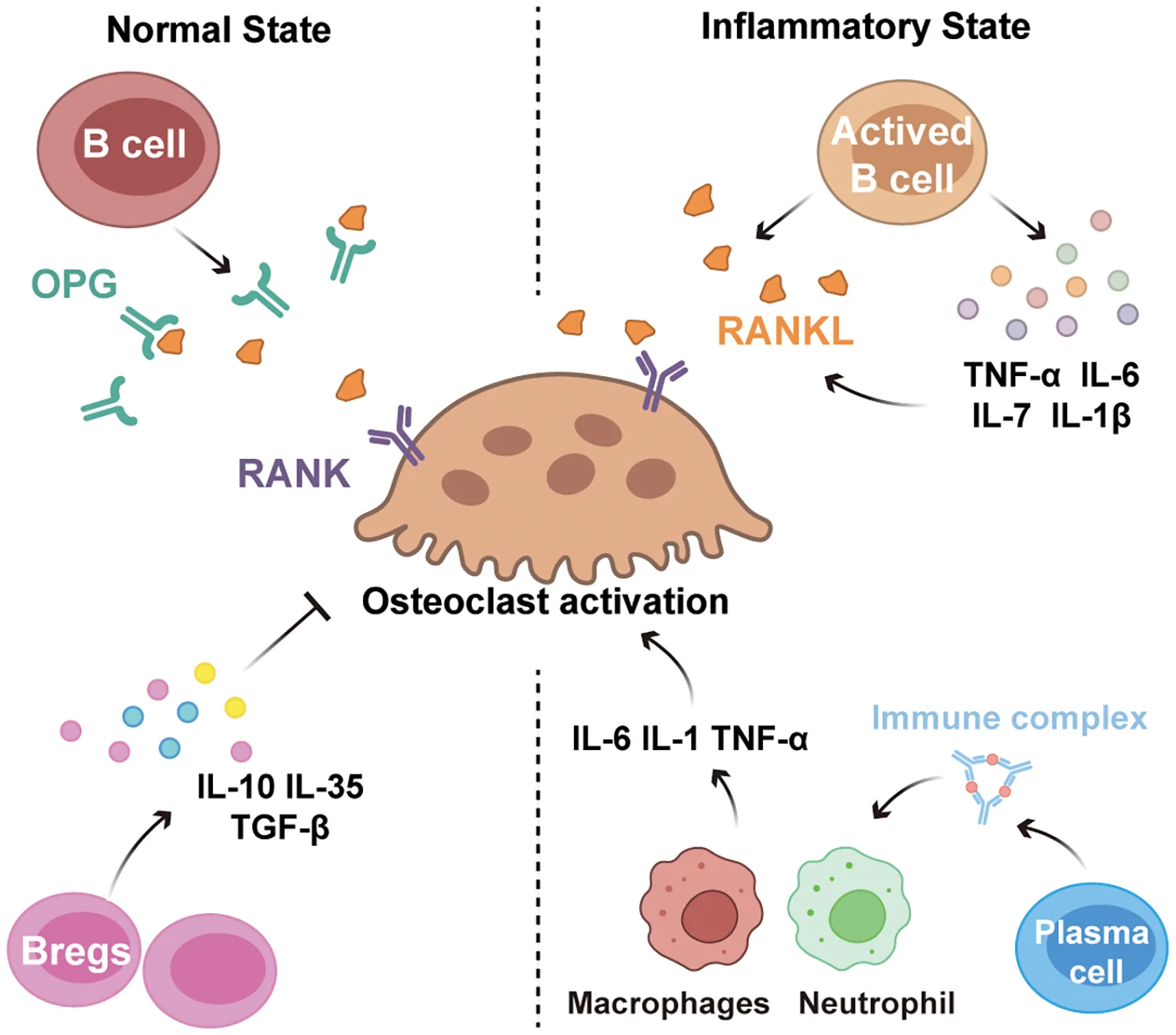
Regulatory mechanisms of B cells and their subsets in bone remodeling. This image illustrates the dual regulatory effects of B lymphocytes on osteoclast activation under physiological conditions and inflammatory pathological microenvironments. Under physiological conditions (left panel), mature B cells primarily exert bone-protective effects. They highly express and secrete osteoprotegerin (OPG), a soluble decoy receptor for RANKL. OPG binds to RANKL and competes with cell-surface receptor RANK on osteoclast precursors for ligand binding, thereby blocking downstream activation signaling. Some regulatory B cell subsets (Bregs) also contribute to bone homeostasis. Physiologically, B cells maintain bone mass by secreting IL-10, IL-35, and TGF-β. Upon exposure to inflammatory cues (right panel), B cells undergo activation and their functional phenotype shifts toward osteoclastogenesis, characterized by robust secretion of RANKL along with TNF-α, IL-6, IL-7, and IL-1β. These pro-inflammatory cytokines collectively establish a microenvironment that is both inflammatory and conducive to bone resorption. In parallel, B-cell-derived plasma cells produce immune complexes that, via Fcγ receptor engagement, stimulate macrophages and neutrophils to release additional IL-6, IL-1, and TNF-α. This supplementary cytokine wave ultimately amplifies osteoclast differentiation and resorptive activity.

### 3.3 Roles of macrophages

Macrophages exhibit remarkable phenotypic plasticity and functional diversity in response to local microenvironmental cues. Within the bone marrow niche, macrophages adopt distinct functional states that exert opposing effects on bone homeostasis: a pro-inflammatory phenotype, characterized by elevated TNF-α, IL-1β, and IL-6 production, promotes osteoclastogenesis and bone resorption; conversely, a pro-repair/tissue-resident phenotype, defined by IL-10, TGF-β, and oncostatin M (OSM) secretion, supports osteogenesis and maintains skeletal integrity (89). While these two polarized states represent functional extremes, a spectrum of intermediate phenotypes exists *in vivo*, and macrophage behavior is dynamically shaped by spatiotemporal microenvironmental cues. This functional framework better captures the dynamic spectrum of macrophage behaviors in the bone microenvironment and is therefore adopted throughout this review. In addition to serving as central players in inflammatory diseases, macrophages also exert critical regulatory roles in bone formation through direct interactions with bone cells (89, 90).

#### 3.3.1 Macrophages and physiological bone homeostasis

Physiologically, macrophages predominantly assume a pro-repair/tissue-resident phenotype that renders them essential for skeletal homeostasis, as they produce IL-10 and TGF-β to maintain tissue balance and counteract bone resorption (56, 91). Multiple studies have shown that macrophage depletion in mice leads to a reduction in osteoblast numbers, markedly impaired bone formation capacity, and a consequent decrease in bone mass (92–94). This demonstrates that macrophages play and essential role in regulating bone formation and maintaining bone homeostasis. Moreover, diminished macrophage abundance correlates with a decline in both the quantity and the osteogenic differentiation potential of bone marrow MSCs (94). In contrast, macrophage expansion via M-CSF supplementation promotes osteogenesis and elevates bone mineral density (95).

The concept of “osteal macrophages (osteomacs)” was introduced in 2008 by Chang et al., who identified resident macrophages in murine and human bone tissues that interact with and regulate osteoblast function both *in vitro* and *in vivo (*90). Subsequent studies have further established that these osteal macrophages support physiological bone remodeling and bone formation (96, 97). These cells exhibit functional characteristics resembling those of the pro-repair/tissue-resident phenotype. However, other studies have suggested that, under specific experimental conditions, high levels of cytokines such as TNF-α and IL-1β in macrophage-conditioned media may inhibit osteogenic differentiation (98, 99). This observation underscores the context-dependent effects of macrophages on bone formation.

Beyond their direct interactions with osteoblasts, macrophages also regulate bone homeostasis through efferocytosis, the process by which apoptotic cells are cleared by phagocytes. In bone, the majority of osteoblasts undergo apoptosis, yet the functional relevance of this process has only recently been explored. Using an inducible murine model to trigger apoptosis in mature osteoblasts, Batoon et al. demonstrated that osteoblast apoptosis stimulates efferocytosis by osteal macrophages, and that this efferocytic activity significantly enhances vertebral bone formation and increases osteoblast precursors in juvenile mice, an effect dependent on macrophages rather than osteoclasts (100). Efferocytic macrophages exhibit a unique transcriptomic signature with expression of factors including VEGFA, suggesting that macrophage efferocytosis actively contributes to postnatal bone growth. Impaired efferocytosis has been associated with aging-related bone loss and various skeletal pathologies, highlighting its relevance to skeletal aging and disease (101).

Macrophage senescence has emerged as a key contributor to age-related bone loss. With advancing age, macrophages undergo cellular senescence and acquire a senescence-associated secretory phenotype (SASP) characterized by elevated secretion of pro-inflammatory cytokines such as IL-6, TNF-α, and IL-1β. Senescent macrophages accumulate in the aged bone marrow niche and contribute to inflammaging, which promotes osteoclastogenesis and impairs osteoblast function (102, 103). Targeting macrophage senescence, either through senolytic agents or by modulating SASP components, represents an emerging therapeutic strategy for age-related bone loss (102, 104).

#### 3.3.2 Macrophage-mediated bone resorption under inflammatory conditions

Upon exposure to inflammatory stimuli, macrophages shift to a pro-inflammatory phenotype, assuming essential functions in the initiation of immune responses and the elimination of microbial pathogens. These activated cells secrete a broad array of inflammatory cytokines, such as IL-1, IL-6, IL-12, IL-1β, and TNF-α (105). This secretory profile contributes to tissue injury and potentiates bone resorption via two distinct routes: directly augmenting osteoclast precursor proliferation and differentiation, and indirectly driving osteoblasts and stromal cells to upregulate RANKL production (56, 106, 107). The net effect of these pathways is the enhancement of osteoclast formation and bone resorption.

Studies have shown that pro-inflammatory macrophages can suppress osteoblast activity through the secretion of factors such as TNF-α and IL-1β. For example, these cytokines inhibit bone formation by downregulating the expression of the key osteogenic transcription factor RUNX2, thereby exacerbating bone loss (108). In addition, pro-inflammatory macrophages have been reported to inhibit osteoclastogenesis in RAW264.7 cells and bone marrow–derived cells through the secretion of IFN-γ and IL-12 (109). This seemingly paradoxical phenomenon suggests that the effects of pro-inflammatory macrophages on osteoclast formation are not fixed (110). This functional flexibility highlights their pronounced plasticity in osteoimmune regulation. Under conditions of persistent inflammation, these effects are often further amplified through cooperative interactions with other immune cell populations.

In RA, pro-inflammatory macrophages respond to the chemokine CCL21 by upregulating their secretion of IL-6 and IL-23, which in turn promotes the polarization of naive T cells toward the Th17 phenotype (111). Th17-secreted IL-17 potently stimulates RANKL production in osteoblasts and stromal cells, leading to heightened bone erosion. In this context, pro-inflammatory macrophages act as critical effectors in inflammatory osteolysis. These cells exert their pro-resorptive influence through three parallel mechanisms: differentiating into osteoclasts, releasing cytokines that destabilize skeletal equilibrium, and cooperating with Th17 and other immune populations.

#### 3.3.3 Macrophage-derived OSM and bone repair

Macrophages also serve as a major source of OSM, which has been demonstrated to facilitate osteogenic differentiation and bone mineralization effectively in both *in vitro* and *in vivo* settings (106). The osteogenic action of OSM is mediated through activation of Runx2 and simultaneous inhibition of sclerostin, the former promoting differentiation and the latter relieving an inhibitory brake on bone formation (112). Furthermore, during fracture repair, OSM augments ALP activity in osteoblasts by engaging the STAT3 pathway (113). Such evidence points to a role for OSM in directing mesenchymal stem cells to adopt an osteoblastic phenotype.

Nevertheless, it should be noted that the origin and activation status of macrophages vary across experimental conditions, resulting in distinct local microenvironments. Consequently, the precise regulatory roles of macrophages in bone homeostasis and the underlying mechanisms remain to be fully elucidated. Moreover, the metabolic state of macrophages, including their preference for glycolysis or oxidative phosphorylation, is increasingly recognized as a critical determinant of macrophage polarization and functional switching (114, 115). The local metabolic milieu within the bone marrow may therefore indirectly regulate the balance between bone formation and bone resorption through this mechanism. Although the role of macrophages in bone metabolism is well established, the precise control of their polarization in different immune contexts remains an important direction for future research.

## 4 Bone lineage cells as active regulators of immune homeostasis

Three major cell types—osteoblasts, osteoclasts, and osteocytes—constitute the cellular basis of the skeleton, with approximate proportions of 5%, 1%, and 90–95%, respectively (3, 87). Osteoblasts drive bone deposition, osteoclasts govern resorption, and osteocytes, as the predominant population, reside largely within the bone matrix. In addition to their established functions in structural support, protection, and mineral metabolism, the skeleton has been increasingly recognized as a source of numerous bone-derived factors (BDFs), encompassing cytokines, metal ions, and hormones (3, 116). These secreted factors participate in the regulation of skeletal homeostasis while also targeting multiple distant tissues, including skeletal muscle, adipose tissue, the liver, pancreas, the CNS, kidneys, and testes (3, 117, 118). Through such systemic actions, they contribute critically to the pathophysiology of various disease states.

As elaborated above, immune cells have been shown to exert significant regulatory influences on bone metabolism. Nevertheless, the skeleton does not function as a mere passive target of immune modulation. Emerging evidence indicates that bone-derived cells, including osteoblasts and osteoclasts, play an active role in steering immune processes. In doing so, they forge a fully functional bidirectional interface that coordinates the activities of the bone and immune compartments. In the following section, we will review in turn the immunoregulatory roles of osteoblasts, osteoclasts, and osteocytes.

### 4.1 Immunoregulatory functions of osteoblasts

Osteoblasts originate from bone marrow–derived MSCs and play central roles in skeletal development, repair, and the maintenance of bone homeostasis. Traditionally, osteoblasts have been viewed as cells that primarily mediate bone formation through the secretion of bone matrix proteins and mineralization-related factors (56). Over the past decade, growing evidence has shifted our understanding. Osteoblasts are now recognized as important modulators within the bone marrow immune microenvironment, rather than mere passive targets of immune regulation (**Figure 3**). Osteoblasts contribute actively to the generation, differentiation, and functional modulation of immune cells by secreting a wide array of cytokines and expressing critical signaling molecules.

**Figure 3:**
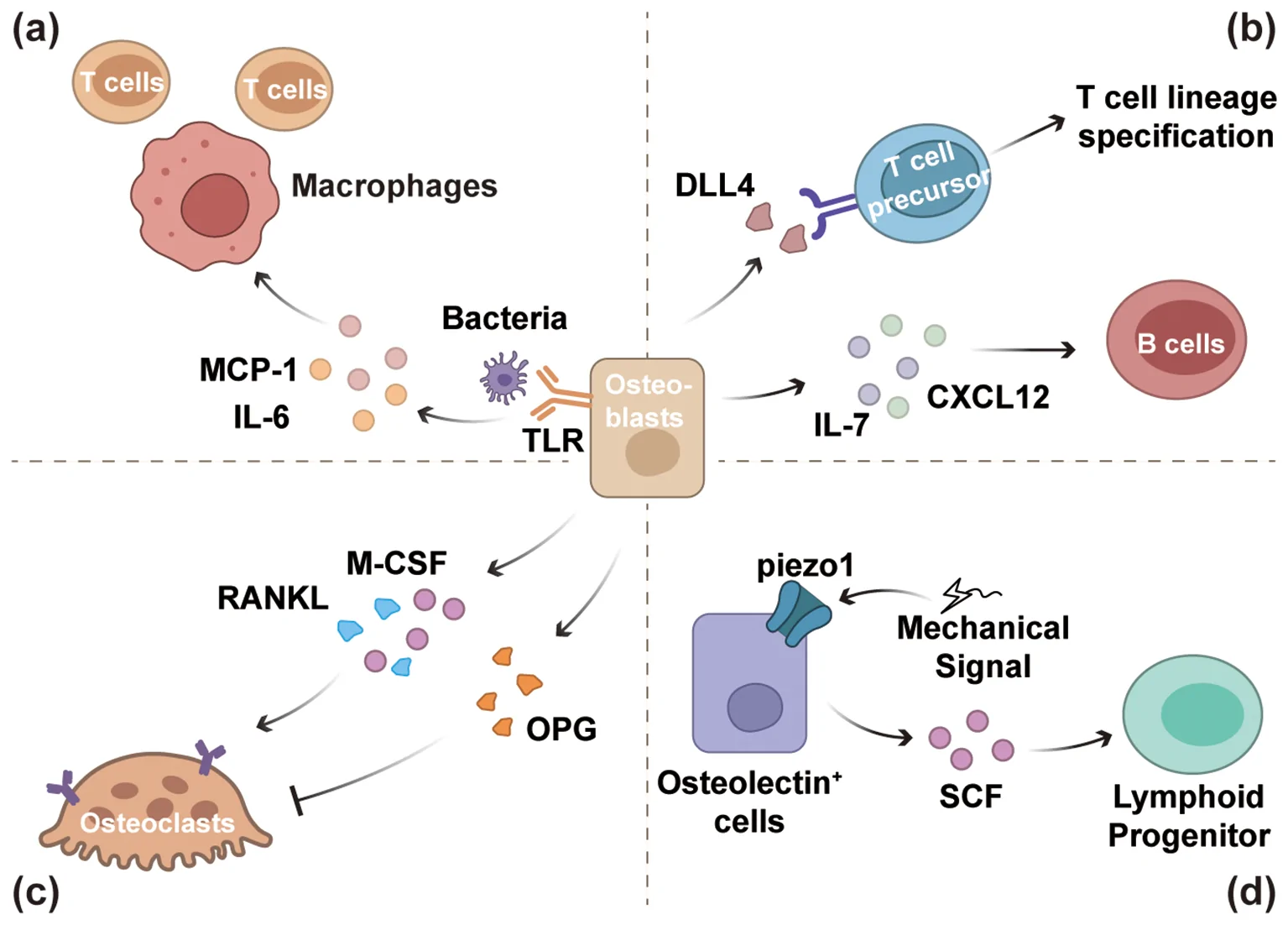
Regulatory network of osteoblasts in immune modulation and bone remodeling. This image systematically illustrates the multiple regulatory functions of osteoblasts in maintaining bone homeostasis and responding to external stimuli. **(a)** In immune regulation, osteoblasts respond to bacterial infection or inflammatory stress via Toll-like receptor (TLR) signaling. Osteoblasts secrete MCP-1 and IL-6 to mediate cellular crosstalk with macrophages. MCP-1 drives the recruitment of macrophages into bone tissues. IL-6 further regulates the activation, phenotypic polarization and differentiation fate of recruited macrophages. **(b)** Osteoblasts modulate the secretory ratio of RANKL to OPG. In concert with macrophage colony-stimulating factor (M-CSF), these factors govern osteoclast differentiation and activation, thereby sustaining the homeostatic equilibrium of bone remodeling under both physiological and pathological settings. **(c)** Osteoblasts act not only as functional cells for bone formation, but also as essential stromal cells constituting the niche for early differentiation of lymphoid progenitor cells. The expression and secretion of DLL4, IL-7 and CXCL12 by osteoblasts provide indispensable signals for lymphoid development. **(d)** Physiological mechanical stimuli include skeletal loading induced by exercise and fluid shear stress. These stimuli directly activate Piezo1 channels on the surface of Osteolectin^+^ cells. Piezo1 activation promotes osteogenic differentiation. It also induces Osteolectin^+^ cells to secrete stem cell factor (SCF), which specifically regulates adjacent lymphoid progenitor cells.

Osteoblasts also function as essential upstream modulators of osteoclastogenesis. They directly influence osteoclast differentiation through the secretion of multiple factors, including RANKL, OPG, M-CSF, WNT5a, and semaphorin 3A (SEMA3A) (116). Additionally, osteoblast-derived WNT4 exerts a dual regulatory effect: it promotes bone formation in an autocrine manner while simultaneously suppressing bone resorption through paracrine signaling (116).

#### 4.1.1 Regulation of immune cells by osteoblasts

Beyond their direct regulation of osteoclast formation, osteoblasts are also indispensable for hematopoietic and lymphoid cell generation in the marrow microenvironment. They furnish vital niche signals for the development and survival of B- and T-cell populations through the production of key molecules such as CXCL12, IL-7, and the Notch ligand Delta-like 4 (DLL4) (6, 119–121).

Osteoblast-derived IL-7 is recognized as an indispensable cytokine for early B-lymphocyte development. This essential role is supported by genetic evidence showing that targeted deletion of Il-7 in osteoblasts leads to a marked reduction in common lymphoid progenitors (CLPs) within the murine bone marrow (121). Similarly, CXCL12 functions as an indispensable chemokine for B-cell development (122), and its conditional ablation in osterix-expressing early osteoblasts has been found to reduce the abundance of B-lymphoid progenitors (119, 123). In contrast, CXCL12 deletion in mature, mineralizing osteoblasts has only a minor impact on B lymphoid progenitor generation (119). These findings suggest that osteoblasts at different stages of differentiation exhibit functional specialization in regulating immune cell development. Further studies have demonstrated that signaling pathways within osteoblasts indirectly influence immune cell generation by regulating the expression of key cytokines such as IL-7 and CXCL12 (124). These pathways include Hedgehog (Hh), mTORC1, and GPCR–Gsα–PKA signaling. Although identified in distinct experimental models and operating independently, these pathways ultimately converge. They coordinately regulate critical niche factors to collectively shape a bone marrow microenvironment that supports immune cell development. In addition, osteoblast-expressed DLL4 can bind to Notch receptors on T cell progenitors, activating the Notch signaling pathway (124). This activation represents a key step in directing T cell lineage commitment.

#### 4.1.2 Osteoblasts and immune responses

In addition to their roles in adaptive immune cell development, osteoblasts can also function as active participants in immune responses within bone tissue. Under pathological conditions like bacterial infection (e.g., *S. aureus*), osteoblasts are capable of sensing pathogens by expressing pattern recognition receptors such as Toll-like receptors (TLRs) (125). Activated osteoblasts secrete a range of inflammatory mediators, including IL-6 and MCP-1, which serve to attract and activate macrophages and T cells at foci of infection (126). Thereby, osteoblasts cooperate with the immune system to clear pathogens. These findings indicate that osteoblasts serve as “signal amplifiers” during bone infection and inflammation. Furthermore, by regulating immune cell recruitment and activation, they actively contribute to local inflammatory responses and associated bone destruction.

#### 4.1.3 Mechanical stimulation and osteoblast-immune crosstalk

Recent studies have further revealed that osteoblasts can translate mechanical stimuli into immunoregulatory signals. This process establishes a functional link between skeletal activity and immune homeostasis. The research group led by Sean Morrison identified a population of osteogenic progenitor cells in the bone marrow, termed Osteolectin-positive (Osteolectin^+^) cells (127). These cells are capable of sensing exercise-induced mechanical forces and transducing mechanical signals through the mechanosensitive ion channel Piezo1. Piezo1 activation promotes osteogenic differentiation and induces the secretion of stem cell factor (SCF). SCF, in turn, supports periarteriolar lymphoid progenitors and enhances lymphopoiesis. This mechanism connects physical activity to immune regulation (127). This discovery suggests that osteoblast-lineage cells can directly couple physiological mechanical signals to immune system function through mechanotransduction pathways. Thus, it significantly expands the conceptual framework of osteoimmunology.

Accordingly, dissecting the molecular pathways through which bone cells influence lymphocyte development both establishes osteoblasts as a key hub connecting the skeletal and immune compartments. It also provides meaningful theoretical perspectives on the mechanisms underlying inflammatory bone pathology and immune-mediated skeletal diseases. Senescent osteoblasts exhibit diminished osteogenic potential and contribute to the aged bone marrow microenvironment that favors bone loss (102).

### 4.2 Immunoregulatory functions of osteoclasts

The principal role of osteoclasts is to mediate bone resorption through the secretion of proteolytic enzymes and the establishment of an acidic environment, which together solubilize the organic and inorganic components of bone. These cells originate from hematopoietic stem cells and are generated by the fusion of mononuclear progenitors under the influence of M-CSF and RANKL (48, 128–130). In addition, osteoclasts transmit signals to osteoblasts and osteocytes by secreting a variety of bone-derived factors, including S1P, ephrinB2, cardiotrophin-1 (CT-1), and semaphorin 4D (SEMA4D) (116, 131). Through these signals, they participate in the regulation of bone formation. Osteoclasts have a well-established role in bone matrix degradation. However, accumulating evidence now reveals that they also engage in bidirectional communication with the immune system through multiple mechanisms.

Osteoclasts can function in a manner similar to antigen-presenting cells (APCs). Although dendritic cells, macrophages, and B cells are classically recognized as professional APCs, osteoclasts have also been shown to possess antigen-presenting capability. Specifically, under inflammatory stimuli, osteoclasts express MHC II and co-stimulatory molecules (e.g., CD80/CD86) to acquire antigen-presenting capacity (29, 48, 132). One well-characterized pro-inflammatory subset is CX3CR1^+^ immunogenic osteoclasts (i-OCLs), which were first identified by the surface marker CX3CR1 and primarily prime TNF-α-producing CD4^+^ effector T cells (132). In parallel, a distinct population of osteoclasts with cross-presentation capacity has been described. These osteoclasts can cross-present antigens and induce the generation of Foxp3^+^ CD8^+^ Tregs, thereby mediating immune tolerance (133, 134). Collectively, these findings demonstrate that osteoclasts exhibit remarkable functional plasticity in immune regulation. This bidirectional immunomodulatory capacity is highly dependent on microenvironmental cues, and positions osteoclasts as key modulators at the interface of bone remodeling and immune homeostasis. Such properties provide novel therapeutic opportunities for intervening in aberrant bone resorption and associated inflammatory diseases, including osteoporosis and chronic inflammatory disorders.

In addition to their resorptive function, osteoclasts secrete a broad spectrum of cytokines that influence immune cell activity. In the steady state, they promote Treg differentiation via IL-10 (48), whereas in chronic inflammation, they produce TNF-α, IL-1, and IL-6, which activate TNF-α^+^ CD4^+^ T cells and exacerbate T-cell-mediated inflammation (48). Moreover, calcium ions (Ca²^+^) and TGF-β released during resorptive activity have been shown to regulate hematopoietic stem cells (HSCs) function (135, 136). In non-inflammatory contexts, osteoclasts recruit and stimulate naive CD8^+^ T cells, leading to the production of IFN-γ, IL-2, IL-6, and IL-10 (134, 137). Increasing evidence indicates that, although osteoclasts are not conventional immune cells, they are capable of promoting inflammatory responses and immune activation. These findings provide new insights into the pathogenesis of autoimmune-associated inflammatory bone diseases and open new avenues for future investigation.

### 4.3 Immunoregulatory functions of osteocytes

Osteocytes are the most abundant cell type in bone tissue, and serve as the primary mechanosensory cells of the skeleton (138–140). Osteocytes secrete cytokines such as OPG and RANKL. They have long been recognized as central regulators of bone homeostasis through their control of osteoblast and osteoclast function. However, with advances in osteoimmunology, accumulating evidence indicates that osteocytes are not merely regulators of bone metabolism. Instead, they play a unique role in the bone–immune network by integrating mechanical stimuli, inflammatory signals, and metabolic status and subsequently secreting signaling molecules (141–143).

Osteocytes are recognized as a major source of RANKL (144, 145). This is exemplified by the observation that mice lacking *Tnfsf11* specifically in osteocytes develop severe osteopetrosis (144), indicating that RANKL produced by osteocytes plays a non-redundant role in maintaining normal bone turnover. Beyond its effects on osteoclast formation, osteocytic RANKL can also interact with dendritic cells and other RANK-positive immune cells in the bone marrow (146, 147), affecting their survival and activation states. These data suggest that osteocytes possess the potential to indirectly modulate immune cell functions.

Osteocyte-derived sclerostin (SOST) serves as a principal negative regulator of the Wnt/β-catenin pathway (3, 148, 149), and its expression levels are now known to be governed by mechanical loading as well as by inflammatory conditions. Systemic inhibition of SOST can markedly enhance bone formation. However, it may also be accompanied by vascular calcification and elevated levels of inflammation-associated factors, increasing the risk of cardiovascular disease (148, 150). These observations suggest that osteocyte-derived factors may serve as critical links between bone metabolism and systemic inflammatory responses.

Under inflammatory or pathological conditions, the functional state of osteocytes can undergo profound alterations (151). Studies have shown that apoptotic or damaged osteocytes secrete chemokines such as IL-6 and RANTES/CCL5 (152, 153). These signals recruit immune cells, including macrophages, to the bone microenvironment and further amplify local pro-inflammatory responses. Additionally, apoptotic or stressed osteocytes release damage-associated molecular patterns (DAMPs) including high-mobility group box 1 (HMGB1) and ATP. These DAMPs, particularly HMGB1, mediate macrophage recruitment to the perilacunar microenvironment and may also promote the recruitment of other immune cells such as dendritic cells, collectively amplifying local inflammatory cascades during pathological bone loss (143, 151, 152, 154, 155). Animal studies have provided additional evidence supporting the link between osteocyte dysfunction and inflammatory bone diseases. For example, knockout of cyclooxygenase-2 (COX-2) in osteocytes or treatment with a COX-2 inhibitor has been shown to attenuate spontaneous osteoarthritis in STR/Ort mice (156). Notably, osteocyte-mediated recruitment of immune cells is unlikely to initiate immune responses *de novo*. Instead, it appears to sustain and amplify the local immune microenvironment under inflammatory conditions.

Collectively, osteocytes function as mechanosensors and central regulators of bone remodeling. Moreover, under inflammatory or pathological conditions, they indirectly participate in shaping the immune microenvironment through the secretion of cytokines and chemokines. Compared with osteoblasts and osteoclasts, osteocytes may not function as direct effector cells that initiate immune responses. Instead, by integrating mechanical, inflammatory, and metabolic signals, osteocytes continuously modulate the bone marrow immune microenvironment and serve amplifying and stabilizing roles within the osteoimmune regulatory network. Together with osteoblasts and osteoclasts, osteocytes form a finely tuned and dynamic osteoimmune regulatory network that determines whether tissues progress toward homeostasis or pathological destruction (143). While current understanding of osteocyte immunoregulation is limited and their precise disease-specific roles remain unclear, this field of study is crucial. It provides a key entry point for understanding osteoimmune imbalance and developing targeted therapies.

## 5 Central bridging roles of cytokines

The preceding sections have systematically delineated the intricate bidirectional crosstalk between immune cells and bone lineage cells at the cellular level. However, the realization of these intercellular interactions ultimately relies on soluble signaling molecules, namely cytokines. The foundation of osteoimmunology lies in the fact that immune cells and skeletal cells share the same bone marrow microenvironment and communicate through common signaling mediators. Cytokines constitute the complex molecular bridges between the immune and skeletal systems. These molecules, produced by immune or bone-derived cells, act on target cells via paracrine or endocrine signaling. They thereby translate immune status changes into adaptive bone remodeling, and vice versa, forming a continuous feedback loop. The preceding sections focused on specific cell types. Here, we shift to a molecular perspective, highlighting key cytokines that serve as central bridges in bone–immune crosstalk and outlining their regulatory modes.

### 5.1 RANKL/RANK/OPG axis

The RANKL/RANK/OPG system constitutes the most definitive and paradigmatic molecular hub in osteoimmune crosstalk (8, 157). RANKL is produced by a variety of cell types, including osteoblasts, osteocytes, and activated T and B lymphocytes (88, 158–160). Collectively, these cellular sources designate RANKL as a bone-derived factor (BDF) that serves as a functional bridge, conveying immune signals to promote osteoclastic bone resorption. Its interaction with RANK triggers osteoclast precursor differentiation and enhances the resorptive efficiency of mature osteoclasts (161).

OPG, a soluble decoy receptor sourced from osteoblasts and various other cells, competitively binds RANKL to block osteoclastogenesis and preserve the steady-state balance of bone remodeling (162). The net outcome of bone resorption, in terms of both intensity and duration, is dictated principally by the RANKL/OPG ratio systemically, not by the absolute abundance of either factor alone. In contexts of dysregulated immunity, exemplified by rheumatoid arthritis, activated T cells substantially elevate RANKL expression, distorting the RANKL/OPG balance and disrupting skeletal homeostasis, which eventually gives rise to pathological bone destruction (11, 13, 14, 17).

### 5.2 Shared cytokines and osteoimmune homeostasis

Beyond RANKL, multiple cytokines are jointly produced by bone cells and immune cells, exerting regulatory effects on both systems. This co-production and dual action underscore the close developmental and functional interconnection between the skeletal and immune systems. IL-6 represents another representative example. Both osteoblasts and immune cells are capable of producing IL-6. This cytokine promotes osteoclastogenesis and drives the differentiation of naive CD4^+^ T cells into Th17 cells, collectively indirectly amplifying a pro–bone-resorptive immune milieu (22, 74, 111, 126). In addition, M-CSF highlights the shared developmental origins of immune cells and bone cells. M-CSF is not only essential for osteoclast differentiation but also serves as a key regulator of monocyte–macrophage lineage development (163, 164). Studies have shown that mutations in the *M-CSF* gene result in defects in the formation of both osteoclasts and macrophages (163, 164). This shared dependence enables cytokine networks to efficiently integrate signals derived from both the immune and skeletal systems.

Moreover, the dynamic balance between pro-inflammatory and anti-inflammatory cytokines ultimately determines the outcome of bone remodeling. As previously discussed, activated immune cells under pathological conditions secrete multiple pro-inflammatory cytokines, including TNF-α, IL-1β, IL-1, IL-6, and IL-17. These cytokines contribute to host defense during acute immune responses. However, their sustained or excessive expression markedly promotes osteoclastogenesis and suppresses osteoblast function, thereby driving bone loss. Importantly, not all immune responses result in bone destruction. In contrast, anti-inflammatory cytokines including IL-10, TGF-β, and IL-4 can maintain bone homeostasis or promote bone repair by directly inhibiting osteoclast differentiation or antagonizing pro-inflammatory signaling. In many chronic inflammatory diseases, this pro-inflammatory–anti-inflammatory balance shifts from a transient disruption to a self-sustaining cycle of inflammation and bone destruction (165–167).

These cytokines operate not in isolation but as an interconnected network. TNF-α and IL-1β synergistically promote osteoclast differentiation and bone resorption, a mechanism well documented in both *in vitro* and *in vivo* models (78, 107). In the T-cell compartment, IL-6 in combination with TGF-β drives the differentiation of naïve CD4^+^ T cells into Th17 cells. These Th17 cells then produce IL-17, which further stimulates RANKL production by stromal cells (21, 168, 169). Thus, a functional link is established between adaptive immunity and bone resorption. IL-1β and TNF-α also directly induce RANKL expression in osteoblasts and stromal cells via distinct but converging signaling pathways (42, 144). Collectively, these synergistic and convergent interactions create a coordinated cytokine network that converges on osteoclastogenesis, rather than operating through independent linear signals.

Collectively, cytokines do not function as isolated signaling molecules, but instead form a complex molecular network that bridges the immune and skeletal systems. Elucidating the patterns of interaction within this cytokine network deepens our understanding of osteoimmune imbalance across different disease. Moreover, it provides a critical foundation for developing precise therapeutic interventions targeting osteoimmune-related disorders.

## 6 Osteoimmune dysregulation in human diseases

Advances in osteoimmunology have yielded fresh insights into the pathogenic mechanisms of disease and simultaneously promoted the evolution of treatment paradigms. Such progress is particularly impactful for a wide array of clinical entities that affect both the skeleton and the immune system, notably including RA, osteoporosis, osteoarthritis, periodontitis, Paget’s disease, multiple myeloma, and bone metastases. Disruption of the sophisticated equilibrium between the skeletal and immune systems can instigate or accelerate the development of inflammatory, metabolic, and malignant bone disorders.

### 6.1 Inflammatory bone diseases

Among inflammatory bone diseases, RA represents one of the most representative disease models in osteoimmunology research. The pathological core of RA is the persistent and aberrant activation of the immune system. This activation disrupts the stability of the bone microenvironment and leads to synovial hyperplasia and periarticular bone destruction. Activated T cells, particularly Th17 cells, produce large amounts of pro-inflammatory cytokines such as TNF-α, IL-6, and IL-17 (21). These cytokines sustain chronic inflammation and strongly stimulate synovial fibroblasts and other bone-related cells to express RANKL (6, 21). This leads to RANKL-dependent osteoclastogenesis and, ultimately, joint bone erosion. Based on this osteoimmune mechanism, biological agents targeting key inflammatory cytokines have emerged as effective therapies for RA. These include anti-TNF-α agents (e.g., infliximab, etanercept) (170), and the IL-6 receptor inhibitor tocilizumab (171, 172). These treatments effectively suppress inflammatory responses and indirectly slow the progression of bone destruction. In addition, the B cell–targeting monoclonal antibody rituximab selectively depletes CD20-positive B cell subsets and shows favorable clinical efficacy in patients with RA (173). This efficacy further confirms the role of adaptive immunity in RA-associated bone damage. Moreover, given the close pathological association between oxidative stress and RA, antioxidant therapies may hold potential therapeutic value (124). Certain probiotics have also been reported to exert beneficial effects in RA by reducing the release of inflammatory cytokines (48, 174).

Periodontitis is another typical condition linked to osteoimmune imbalance, and is essentially an oral bone disease driven by local immune dysregulation. Mechanistically, periodontal pathogens provoke host defenses and activate periodontal ligament cells as well as macrophages, leading to IL-6 production (175). Through this inflammatory cascade, Th17 and exFoxp3 Th17 cells accumulate and produce IL-17, IL-1, and TNF, which together upset the local RANKL/OPG ratio and potentiate osteoclast-mediated bone degradation. Ultimately, these pathological events lead to the resorption of alveolar bone and subsequent tooth loss (6, 176). In terms of therapeutic translation, Ustekinumab has been reported to alleviate periodontitis via inhibition of Th17 activation (177), and the IL-6 receptor antagonist Tocilizumab has shown effectiveness in controlling periodontal inflammation among RA patients (178). Therefore, a dual strategy combining osteoimmune-modulating agents with robust antimicrobial measures may represent a viable approach to prevent pathogen-associated bone damage.

Ankylosing spondylitis (AS) exemplifies a distinct osteoimmune paradigm. Unlike rheumatoid arthritis or periodontitis where bone resorption predominates, AS features a paradoxical coexistence of osteoclast-mediated periarticular erosion and pathological new bone formation (enthesophytes and syndesmophytes) (179). The IL-17/IL-23 axis is a core driver of local inflammation in AS and contributes to dysregulated bone metabolism, in part by modulating Wnt/β-catenin pathway activity and thereby influencing osteoblast function (180–182). Although TNF inhibitors and IL-17 antagonists (e.g., secukinumab) have demonstrated clinical efficacy, the molecular switch governing the transition from bone resorption to pathological ossification remains incompletely understood.

### 6.2 Metabolic bone diseases

Recent studies have demonstrated that the immune system plays an important regulatory role in the initiation and progression of osteoporosis (11). Postmenopausal osteoporosis (PMO) serves as a key example of osteoimmunology (88). Estrogen deficiency is considered the primary cause of this condition (87). In addition to directly affecting bone cells, estrogen deficiency exacerbates bone loss by disrupting immune homeostasis (183–185). The central mechanism involves estrogen-dependent alterations in the function of key immune cell populations. The differentiation, proliferation, and cytokine secretion profiles of T cells, including CD4^+^ T cells, CD8^+^ T cells, regulatory T cells, and Th17 cells, are shifted toward a pro-osteoclastogenic phenotype (64, 186, 187). Plasma cells differentiated from B cells can also secrete autoantibodies that promote osteoclast differentiation (87). In addition, estrogen contributes to bone homeostasis by regulating macrophage cytokine production (e.g., IL-1, IL-6, TNF-α, interferon-γ) through estrogen receptor–mediated signaling pathways, including PI3K and NF-κB (87, 105). Collectively, these immune alterations enhance osteoclast activity while suppressing osteoblast function, ultimately driving bone loss.

Aging itself drives a distinct layer of osteoimmune dysregulation that directly aligns this review with the Research Topic on cellular senescence and aging. Aging leads to the accumulation of senescent osteocytes and mesenchymal stromal cells within the bone marrow niche. These cells develop a SASP dominated by pro-inflammatory factors such as IL-6 and TNF-α. Notably, senescent osteocytes also show elevated RANKL expression. Consequently, this establishes a persistent low-grade inflammatory state known as inflammaging, which provides a favorable microenvironment for osteoclast differentiation and activation (102, 103, 188). Concurrently, immunosenescence remodels the T-cell pool by expanding CD28^-^ effector memory T cells that produce excessive IFN-γ and TNF-α, while diminishing naive T-cell output (66, 67). These senescence-associated alterations represent a core pathogenic mechanism in senile osteoporosis, and drive bone loss partially independent of estrogen deficiency. Accordingly, the pharmacological elimination of senescent cells or modulation of SASP components constitutes a promising therapeutic frontier in osteoimmunology (102, 104, 189).

From a therapeutic perspective, the RANKL inhibitor denosumab has been widely used for the treatment of osteoporosis in postmenopausal women (190, 191). Clinical trials have shown that denosumab significantly increases bone mineral density at multiple skeletal sites and exhibits superior efficacy compared with traditional oral bisphosphonates (192). However, the occurrence of rebound bone loss following treatment discontinuation highlights the systemic costs associated with long-term and potent suppression of key osteoimmune pathways. The underlying mechanism involves the accumulation of osteoclast precursors during sustained RANKL inhibition. Upon withdrawal of inhibition, bone resorption rapidly rebounds. This phenomenon indicates that osteoimmune-targeted therapies should balance therapeutic efficacy with long-term safety (193, 194).

Despite their efficacy, current osteoimmune-targeted agents share notable clinical caveats. Discontinuation of denosumab leads to rapid rebound of bone resorption and elevated risk of vertebral fractures. The accumulation of osteoclast precursors during long-term RANKL blockade is widely regarded as the core underlying mechanism (193, 194). Romosozumab, despite potent anabolic effects, carries a boxed warning for major adverse cardiovascular events (150, 195, 196). Moreover, prolonged TNF-α or IL-6 blockade increases susceptibility to opportunistic infections. These limitations underscore the need for optimized sequential regimens and patient-specific risk stratification.

### 6.3 Tumor-associated bone diseases

Breast, prostate, and multiple myeloma tumors are among the malignancies with a well-recognized tropism for bone (124).However, they exhibit fundamentally distinct patterns of bone involvement. Breast cancer bone metastases are predominantly osteolytic, driven by tumor-derived parathyroid hormone–related protein (PTHrP) and IL-6, which stimulate RANKL expression and osteoclast activation (197, 198). In contrast, prostate cancer metastases are predominantly osteoblastic, accounting for over 90% of its osseous metastases, and are characterized by aberrant pathological new bone formation rather than dominant osteolytic lesions (199). Multiple myeloma, a plasma cell malignancy that develops almost exclusively within the bone marrow, generates devastating osteolytic bone destruction through direct stimulation of osteoclastogenesis and suppression of osteoblast differentiation (200). Despite these distinct pathological features, a common theme across these diseases is the disruption of bone-immune homeostasis within the marrow microenvironment, which creates a permissive niche for tumor progression and bone pathology. At the same time, tumor-associated immunosuppressive states, such as the accumulation of regulatory T cells and myeloid-derived suppressor cells, weaken antitumor immune responses. This immunosuppression thereby creates a niche tumor survival and expansion within bone (201–203).

Accordingly, targeting RANKL has become a central strategy in both the prevention and treatment of metastatic bone disease. Denosumab and other RANKL inhibitors have been shown to effectively disrupt this pathological feedback loop (204, 205). Consequently, these agents are routinely used in clinical practice to minimize bone-related complications among individuals with end-stage cancers. Despite significant and sustained efficacy over months, the therapeutic effects of these agents rapidly diminish upon discontinuation (193, 206). This indicates that optimal treatment strategies require further investigation and refinement. Moreover, the heterogeneity of osteoimmune dysregulation across bone-related diseases requires therapies that are both disease-specific and systemically safe in the long term. The goal of such targeted approaches is to control progression while preserving the physiological functions of the skeletal and immune systems.

## 7 Conclusion and future perspectives

Osteoimmunology, an emerging and rapidly evolving discipline, has revealed a complex bidirectional regulatory network that links the skeletal and immune systems. From the perspective of inter-organ crosstalk, this bone-immune interface demonstrates how skeletal tissue integrates and transmits systemic signals, establishing the skeleton as a central hub in whole-body homeostasis rather than a passive target organ. This review delineates the molecular basis of crosstalk between T cells, B cells, macrophages and bone cells (osteoblasts, osteoclasts, osteocytes) via shared cytokines and signaling cascades. Under physiological conditions, these interactions maintain skeletal and immune homeostasis. In contrast, under pathological conditions they jointly drive the progression of a wide range of diseases, including rheumatoid arthritis, periodontitis, osteoporosis, and tumor-associated bone metastasis. A deeper understanding of osteoimmune interactions provides novel insights into disease pathogenesis, thereby establishing a translational framework for developing therapies against inflammatory bone diseases, osteoporosis, and tumor-related bone disorders.

Despite substantial advances, the field of osteoimmunology continues to face significant challenges and limitations. Osteoimmune mechanisms may exhibit marked heterogeneity across different diseases, such as rheumatoid arthritis and osteoporosis, or even across distinct stages of the same disease. A major task for future research is to delineate these disease-specific immune–bone interaction networks. This will provide a solid theoretical basis for advancing precision medicine. Moreover, key cellular players, including osteoclasts and B cells, often exert dual or even multiple roles in osteoimmune regulation. Their functions are highly context-dependent and dynamically shaped by the local microenvironment. This functional plasticity substantially increases the complexity of targeted therapeutic interventions. The crosstalk between immune and bone cells is intricate and dynamic. Future studies will continue to explore the fine regulatory mechanisms governing bone–immune crosstalk, with the aim of identifying new therapeutic opportunities for refractory skeletal and immune-related diseases.
